# Strategies to facilitate integrated care for people with alcohol and other drug problems: a systematic review

**DOI:** 10.1186/s13011-017-0104-7

**Published:** 2017-04-07

**Authors:** Michael Savic, David Best, Victoria Manning, Dan I. Lubman

**Affiliations:** 1 Turning Point, Eastern Health, 54-62 Gertrude St, Fitzroy, VIC 3065 Australia; 2 Eastern Health Clinical School, Monash University, Level 2, 5 Arnold Street, Box Hill, VIC 3128 Australia; 3 Department of Law and Criminology, Sheffield Hallam University, Heart of the Campus Building, Collegiate Crescent, Collegiate Campus, Sheffield, S10 2BQ UK

**Keywords:** Integration, Health services, Alcohol, Drugs, Dependence, Substance use disorders, Implementation, Co-occurring issues

## Abstract

**Background:**

There is a growing body of research highlighting the potential benefits of integrated care as a way of addressing the needs of people with alcohol and other drug (AOD) problems, given the broad range of other issues clients often experience. However, there has been little academic attention on the strategies that treatment systems, agencies and clinicians could implement to facilitate integrated care.

**Methods:**

We synthesised the existing evidence on strategies to improve integrated care in an AOD treatment context by conducting a systematic review of the literature. We searched major academic databases for peer-reviewed articles that evaluated strategies that contribute to integrated care in an AOD context between 1990 and 2014. Over 2600 articles were identified, of which 14 met the study inclusion criteria of reporting on an empirical study to evaluate the implementation of integrated care strategies. The types of strategies utilised in included articles were then synthesised.

**Results:**

We identified a number of interconnected strategies at the funding, organisational, service delivery and clinical levels. Ensuring that integrated care is included within service specifications of commissioning bodies and is adequately funded was found to be critical in effective integration. Cultivating positive inter-agency relationships underpinned and enabled the implementation of most strategies identified. Staff training in identifying and responding to needs beyond clinicians’ primary area of expertise was considered important at a service level. However, some studies highlight the need to move beyond discrete training events and towards longer term coaching-type activities focussed on implementation and capacity building. Sharing of client information (subject to informed consent) was critical for most integrated care strategies. Case-management was found to be a particularly good approach to responding to the needs of clients with multiple and complex needs. At the clinical level, screening in areas beyond a clinician's primary area of practice was a common strategy for facilitating referral and integrated care, as was joint care planning.

**Conclusion:**

Despite considerable limitations and gaps in the literature in terms of the evaluation of integrated care strategies, particularly between AOD services, our review highlights several strategies that could be useful at multiple levels. Given the interconnectedness of integrated care strategies identified, implementation of multi-level strategies rather than single strategies is likely to be preferable.

**Electronic supplementary material:**

The online version of this article (doi:10.1186/s13011-017-0104-7) contains supplementary material, which is available to authorized users.

## Background

People with alcohol and other drug (AOD) problems, and especially those with high levels of problem severity or who may be diagnosed as having a substance use disorder, access services with multiple needs or issues beyond their AOD use [1]. These can include mental health, medical, housing, unemployment, education and training needs, as well as issues with criminal justice and social services [2–4] and potentially intimate partner violence [5]. Unmet psychosocial needs can result in treatment drop out, highlighting the need for a holistic approach to treatment [6].

However, AOD services have not always been equipped to address the multiple and complex needs of clients in-house. Treatment for people with AOD problems and co-occurring issues has historically fallen into two categories; serial treatment and simultaneous/parallel care [7]. Serial treatment is where care for AOD problems is delivered before or after care for other problems in separate systems of care [7], in a sequential referral model. In contrast, simultaneous/parallel care occurs in two separate and non-coordinated systems at the same time [7]. Due to a lack of connectedness within and between systems, coordination of care and navigation of complex systems is often left to clients [8]. This has led to calls for responses that guide treatment systems, agencies and clinicians in how best to implement integrated care between AOD services and between AOD and non-AOD services [4, 8, 9]. In this article, we review the academic literature to examine what approaches have been trialled to facilitate integrated care, and propose a model for implementing integrated care strategies.

### What is integrated care?

While a detailed review of the conceptual roots and definitions of ‘integrated care’ is beyond the scope of this article, it is important to reflect on definitions briefly. A recent literature review identified around 175 definitions related to integration in a health system context and highlighted a considerable degree of conceptual murkiness [10]. For instance, often the terms ‘integration’, ‘integrated care’ and ‘integrated service delivery’ are used interchangeably and definitions vary in relation to the scale at which they focus on, ranging from macro-level systems definitions to micro-level clinical conceptualisations [10]. Despite the lack of a universal definition and the interchangeable use of terms, continuity of care, coordination and the adoption of a person-centred approach are often key features of many existing definitions [10]. For instance, integrated care is commonly considered to be a means of delivering health and social care services by coordinating the efforts of services that otherwise act as single units [11] in order to respond more effectively and efficiently to the multiple and complex needs of patients [12]. In a highly cited conceptual article, Kodner and Spreeuwenberg [12] offer a definition that spans multiple levels, and thus, is particularly useful:

Integration is a coherent set of methods and models on the funding, administrative, organisational, service delivery and clinical levels designed to create connectivity, alignment and collaboration within and between the cure and care sectors… (pg 3)

Authors have attempted to distinguish between different types of integration, including horizontal and vertical integration [10]. According to Leichsenring, [11] horizontal integration aims to link parts within a single level of care. In contrast, vertical integration attempts to coordinate the responses of different levels of care (e.g., primary, secondary and tertiary) [11]. However, there is also conceptual ambiguity and debate around the helpfulness of these further differentiations. In the context of people with AOD and other co-occurring problems, we would argue that it is important to conceptualise integration in two ways: 1) coordination between AOD services, such as detoxification and residential rehabilitation, which may ensure greater continuity of care within a client’s treatment journey (within the AOD system); 2) coordination between AOD and non-AOD services such as housing, mental health and community health to ensure multiple needs are met (between systems).

Irrespective of the type of integration and similar to other definitions [10], Kodner and Spreeuwenberg [12] propose that the goal of integrated care is broadly to:

…enhance quality of care and quality of life, consumer satisfaction and system efficiency for patients with complex, long term problems cutting across multiple services, providers and settings [12] (pg 3)

While most clients with complex AOD problems and multiple needs would harbour similar aspirations in terms of receiving quality care that enhances their quality of life, it is important to note that integrated care may not be every client’s preferred way of meeting these aspirations. In certain population groups where concerns about anonymity, privacy, or stigma may be present, people may wish to access services in a serial or simultaneous way.

It is also noteworthy that one of the goals of integrated care relates to improving ‘quality of life’, which is also a commonly held goal of clients and AOD treatment services irrespective of whether clients want to cease or reduce harms related to their AOD use [13]. Indeed harm-reduction strategies, such as opiate substitution therapy and needle and syringe programs, are successfully integrated into AOD treatment systems in many countries and there have been calls for greater integration [14–16].

### Evidence of the effectiveness of integrated care for people with AOD problems

The major focus of the literature on the effectiveness of integrated care for people with AOD problems has been around the integration of AOD and mental health care. With some exceptions (e.g., [17]), systematic reviews of empirical studies generally report that clients receiving integrated care report improved AOD and/or mental health outcomes [18, 19] and higher satisfaction with treatment than clients receiving standard treatment [20].

Similarly, randomised trials evaluating the effectiveness of the integration of AOD and medical care have found higher rates of abstinence from AOD without adding significant additional costs amongst clients receiving integrated care [21–23]. Further evidence suggests that integrated medical and AOD care may confer long-term benefits in terms of medical, wellbeing and functioning outcomes six months after treatment [24] and up to nine-years post-treatment entry [25]. A meta-analysis of integrated AOD and pregnancy, parenting or child services found that integrated care was associated with reductions in AOD use but further research is needed to ascertain whether outcomes are better than non-integrated care [26].

The effectiveness of integration between AOD and social services (e.g., housing, employment, welfare etc.) is less well studied than the two aforementioned areas. Having said this, a clinical trial of coordinated case-management (integrated care) to treat AOD issues amongst clients of welfare agencies in the United States reported positive results [27]. The study found that clients who received integrated care utilised more services than standard care clients, and had significantly higher abstinence rates. While on the whole, the evidence seems to support integrated care between AOD and non-AOD agencies, there is a need to better understand the mechanisms through which integrated care works, and how it can be improved in different contexts.

### General strategies to foster integrated care

Kodner and Spreeuwenberg [12], who viewed healthcare at macro and micro levels, highlighted a continuum of interconnected strategies that can foster integrated care in general at five interconnected levels, including: (i) funding, (ii) administrative, (iii) organisational, (iv) service delivery, and (v) clinical (see Additional file 1). At the funding level, the way health and welfare services are funded can be influential in terms of how well services collaborate and work together [12]. For instance, if services directly compete for funds through competitive tendering processes, they may be less likely to collaborate than if they received block funding. Likewise funding by discrete episodes of care, may mean there is less of an emphasis on complex needs, which may require a longer time to address.

The administrative level refers to government regulatory and administrative departments, which play a role in integrated care through their influence over treatment system design and stewardship [12]. Inter-departmental planning, for example, may result in better integrated systems that are able to accommodate the multiple needs of clients. At the organisational level, a number of strategies can be employed by AOD and other agencies to improve integration [12]. These can include formally and informally networking, collaborating with other agencies, and joint working. Joint working strategies can include multi-agency teams and co-location of staff in which staff from one agency are placed in another agency for a day a week for instance.

Service delivery models can also constrain or encourage integrated care [12]. How staff are trained, deliver care, relate to their clients and colleagues, and how they work together has an impact on clients' experience of integrated care. Staff utilising a case-management model of service delivery may be well-placed to deliver integrated care. At the micro or clinical level, clinical practices, tools and decisions impact on whether clients’ needs are met in an integrated fashion [12]. For example, a common professional language and tools (such as screening and assessment tools), as well as practice standards can facilitate integrated care and embed a holistic way of working in everyday practice.

Kodner and Spreewenberg [12] proposed a typology based on the literature and direct experience, which has been widely cited by others in the integrated care arena. The applicability of these strategies and evidence of their effectiveness in providing care to clients with AOD and other problems however is unclear. This means that AOD services and clinicians that are attempting to provide care to clients with multiple issues have little to guide them in deciding which strategies might be most useful to implement. This lack of specific guidance also poses a problem for policy makers and funders when they are considering action plans for enhancing integrated care. Given this, we examine what works in terms of integrated care by performing a systematic narrative review of the literature. Specifically we address the following questions:

1. What factors/strategies contribute to or improve integration between AOD services?
2. What factors/strategies contribute to or improve integration between AOD and non-AOD services, such as mental health, primary care, housing and other services?

## Methods

In 2014, we conducted a systematic review of the literature using the Preferred Reporting Items for Systematic reviews and Meta-Analysis guidelines [28]. We searched the major academic literature databases for articles that evaluated strategies that contribute to integrated care in an AOD context. These included Scopus, Cochrane, PsychInfo, Web of Science, and CINAHL. We used the search strategy outlined in Table 1 to identify potential articles for inclusion, along with a hand search of articles included in reference lists.

**Table 1:** Search strategy

Search term 1: AOD	Search term 2: Service	Search term 3: Integrat*
Alcohol	Treatment	Partnership
“Illicit drugs”	Provider	Collaborat*
Cannabis	Agency	Coordinat*
Heroin	Program*	Comprehensi*
Methamphetamine	Rehab*	Seamless*
“Substance use”	Detox*	“Transmural care”
“Substance abuse”	Withdrawal	Consolidation
“Drug depend*”	Pharmaco*	“Health care package”
“Addict*”	“Primary care”	“Care network*”
Ice	“Behavioral care”	“Joint venture*”
“Substance misuse”	“Mental health care”	“Interdependence”
“Alcohol and other drug”	“Housing service*”	Continuity
“Drug and alcohol”	“General Practice”	“Inter-agency”
	“Social care”	“Integrated care”
	“Legal services”	“Linkage*”

*Wildcard searches used to retrieve variations on a distinctive word stem or root

“”Exact term searches

(Variants of Search Term 1) AND (Variants of Search Term 2) AND (Variants of Search Term 3)

Although the search logic remained the same across all databases, we modified the search terms used to optimise the search results in particular databases.

Articles were included if they reported on an empirical quantitative, qualitative or mixed-methods study to evaluate or describe the implementation of strategies to facilitate integrated care for people with AOD and other co-occurring problems. Articles were excluded if they:

- focussed on integration between services not involved in AOD care (e.g., integration between medical and mental health services were excluded)
- were published in languages other than English
- were published prior to 1990
- were not published in peer-reviewed journals
- did not evaluate an integrated working strategy/factor/intervention (e.g., letters or commentary pieces were excluded although reviews were included)

**Fig. 1:**
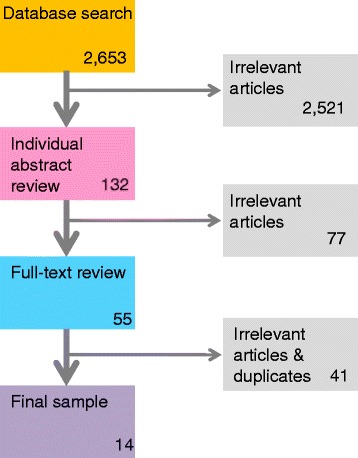
Article selection process

In the first instance, titles and abstracts were screened for suitability for inclusion by the first two authors. All potentially suitable articles were saved in an Endnote library, where duplicates were removed. The remaining articles were read in full and were discussed by the first two authors. Decisions about inclusion were discussed in relation to the aforementioned criteria, and consensus achieved. Any articles that were not suitable were deleted at this point, and a final sample of 14 articles was achieved. Figure 1 outlines the process of article selection.

Information on study characteristics, integrated working strategies and study limitations were recorded for each article in a Microsoft Excel database. This enabled us to appraise the quality of studies in order to make suggestions. We then synthesised the integrated working strategies utilised or recommended in articles according to the levels outlined by Kodner and Spreeuwenberg [12]. This involved categorising integrated working strategies identified according to their level on the continuum of care.

## Results

Additional file 2 provides a summary of the studies included and the integrated working strategies identified in each. As with the literature on the effectiveness of integrated AOD care, most studies were focused on evaluating or reflecting on strategies to facilitate integrated care between AOD and non-AOD services. Studies mainly focussed on strategies at the clinical, service delivery and organizational levels, with many evaluating a combination of strategies at different levels. Most studies were cross-sectional surveys, case-studies, mixed-method studies or qualitative studies from North America, Europe and Australia, and few experimental or longitudinal studies were found. Similarly, very few studies used measures to evaluate the impacts of implementing integrated working strategies on treatment outcomes for clients.

**Fig. 2:**
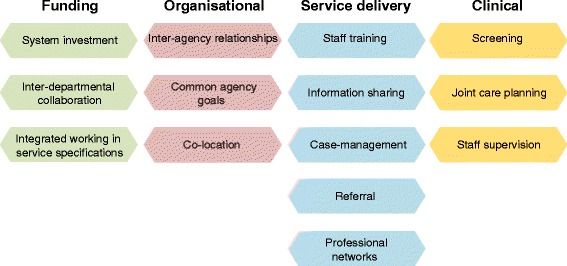
Main strategies by level

The review identified a number of interconnected strategies to enhance integrated working at a number of the levels proposed by Kodner and Spreeuwenberg [11], although no administrative level strategies were identified. The main strategies are illustrated in Fig. 2:

## Level 1: funding

Some studies identified or recommended integration strategies at a funding level. These included system investment, inter-departmental collaboration, and ensuring that integrated working is included in service specifications.

### System investment

Urada et al. [29] conducted surveys with primary care service staff and in-depth qualitative interviews with managers, clinicians, and clients about barriers and facilitators of integration between primary care and AOD services in California. Problems with how primary care clinicians were reimbursed for providing AOD care were identified. For instance, under the current Medi-Cal system (California’s universal health care scheme), providers cannot bill for treatments concurrently provided for physical health and behavioural health issues on the same day, creating a disincentive for providing AOD care. Similarly the range of clinicians eligible for reimbursement for AOD problems under Medi-Cal was limited and did not include AOD counsellors or marriage and family therapists. Participants in this study suggested using policy to enable same day billing, and expand the range of providers who can bill for AOD services under Medi-Cal.

Urada et al. [29] also found that a lack of AOD services and supports in the community was perceived to be a barrier to integration. For instance, a lack of withdrawal/detox and residential treatment options in the community meant that primary care providers had limited or no options to refer clients with severe AOD problems. Injecting funds and resources into the specialist AOD system to increase capacity to meet demand is likely to not only be beneficial for within AOD system integration, but also for integration between the AOD and other systems [29]. This is the idea that increasing the capacity of the AOD system to see new clients would mean that non-AOD agencies would have more and better equipped AOD agencies that they could refer to, and more staffing resources and capacity for effective integration.

### Inter-departmental partnerships within government

Partnerships between different government departments were also found to promote organisational collaboration. This can occur through joint planning and joint funding, in which multiple government departments contribute to funding services and programs of mutual relevance [30]. This opens up access to greater resources for agencies, but also ensures that integrated working is built in to service specifications and funded accordingly.

### Integrated working in service specifications

Another funding level strategy identified was the inclusion of integrated working in service specifications. One government department can be responsible for funding different types of agencies involved in the delivery of an integrated program, with a stipulation that services are delivered in partnership [30]. For example, AOD and non-AOD agencies could be funded from the same government department to provide integrated homeless AOD services. By explicitly including integrated working in service contracts, funders are effectively saying that integrated working is core business. Similarly, in the context of integration between AOD and intimate partner violence services, Timko et al. [31] recommend that programs be funded to address co-occurring problems. One of the key recommendations from Lee et al.’s [30] identification of collaborative care models is that:

Government, organisational, and clinical leadership is needed to promote and reward collaborative practice and establish incentives to facilitate integrated care. (pg 343)

The strategies that we will now discuss all require resources, incentives and leadership if they are to be prioritised as core business and implemented effectively.

## Level 2: organisational

The main strategies that studies identified at the organisational level were the development of inter-agency relationships; shared organisational purpose, values and priorities; and the co-location of services.

### Inter-agency relationships

Two studies emphasised the role of cultivating, fostering and maintaining positive inter-agency relationships [30, 32]. Sword et al. [32] surveyed 270 agencies to examine factors that promote effective linkages between agencies offering treatment services for women with AOD problems and other agencies in Canada (e.g., mental health, health care and social services). They found that strong inter-agency relationships underpinned the success of many integrated working strategies. For instance, agencies were most likely to receive referrals, share information, and engage in joint programming and consultation if clinicians perceived the overall quality of the inter-agency relationship as good. Perceptions of partner agency friendliness and responsiveness to clients were also found to be linked to whether joint programming and consultation occurred.

Sword et al. [32] also mapped the networks of agency partnerships and found large clusters of agencies around four central agencies that had a greater number of partnerships to other agencies in their network. As Sword et al. [32] explained, strategies could be employed to ensure that highly connected agencies play a leadership role in bringing poorly connected agencies into the network.

Scharf et al. [33] found that memoranda of understanding (MOU) were the most common way of formalising relationships (65%) between primary care and behavioural health care (including AOD) services, followed by formal contracts (21%), letters of commitment (12%), and unspecified arrangements (1%). The fact that services used a range of different methods to formalise relationships suggests that the approach taken may depend on the agency context and requirements.

Importantly however, formalising relationships was found to ensure agency commitment and partnership accountability [30]. Documenting expectations and partnership goals at the outset enabled agencies to monitor and measure their progress against goals [30]. Process documentation also helped to prevent the loss of relationships developed by staff when they leave an agency.

### Shared organisational purpose, values and priorities

In a study of collaborative practices between child welfare and AOD agencies, Drabble [34] identified that consensus and agreement on shared values and priorities (e.g., about the importance of addressing AOD issues) is required first, before this can be translated into formal polices and integrated working practices. These formal integrated working policies and practices are likely to be further enhanced by outlining clear agency roles and responsibilities [35, 36].

However, before this can occur, agencies need to be aware of their own values, philosophy and strategic vision. Many sectors of care aspire to provide client-centred care [37], and so client-centred care can be a common point of consensus from which agency and worker relationships are developed.

Other studies suggest that organisational values don’t necessarily have to be in total alignment if participating agencies/groups have practical, complementary goals that individual agencies cannot achieve alone [35]. Vanderplaschen et al. [36] describe this in terms of collaboration needing to be a ‘win-win’ situation for agencies involved, and that there may be a need to clearly differentiate services and roles to also maintain independent identities. Integrated working relationships that are based on complementary goals, may end when goals are achieved, but as Lindholm et al. [35] found, they can be re-activated when the need arises.

Two studies mentioned using a neutral convener as a way of enhancing integration in coalition-type situations [35, 36]. The need for this presumably arises from the experiences of Lindholm et al. [35] of conflict between partners in community coalitions around issues like decision making, distribution of resources and program content. A neutral coalition convener is someone who is perceived by member agencies to be neutral (or not have a stake in the goals of one agency over another) and who can help to resolve disputes between members of the coalition.

Establishing a partnership advisory group was another governance structure option identified [30]. This was not only found to be a forum in which to review the performance of the partnership against pre-determined indicators, but also an avenue through which disputes and collaboration barriers could be resolved. Consumer and carer input into this group as well as other integration activities is also likely to be beneficial in ensuring that activities are grounded in client expectations and experiences.

### Co-location of services

Gurewich and colleagues [38] conducted a series of case studies to investigate strategies that promote effective integration of AOD services in community health centres (CHCs). Gurewich et al., [38] found that all CHCs studied had behavioural health (BH) providers (social workers mostly) embedded within their service or located close by. They found that having BH providers co-located in CHCs increased the likelihood that primary care providers would screen for AOD and other BH care needs. Co-location was also found to facilitate clients’ transition from primary care to specialist behavioural health care. Despite the potential for increased access [38, 39], co-location also presented challenges to BH clinicians who felt that it was difficult to balance the need to provide urgent care to primary care clients who had been referred to them, as well as attending to their own existing caseloads. One way that this was overcome by some sites was by allocating one dedicated BH provider to handling urgent care referrals from primary care providers. This allowed other BH workers to provide care to existing clients.

Scharf et al. [33] reported on the co-location from the other perspective, describing the characteristics and program features of services where primary care was integrated in BH settings in the United States. One of the implementation barriers to co-location at the start of the study was a lack of physical space. Along with the challenges of coordinating multiple providers, a lack of physical space was also a barrier noted by Sylla et al. [39]. At follow-up a year later however, physical space issues had largely been resolved, illustrating that some challenges associated with embarking on co-location can be expected at start-up but can also be addressed over time.

## Level 3: service delivery

Staff training, information sharing, case-management, referral and the development of staff inter-professional networks emerged as key strategies at the service delivery level. Most of these were found to be underpinned by strong inter-agency relationships.

### Staff training

Training of clinicians (and in some cases managers) was one of the strategies identified by a number of studies to improve integrated working. Lee et al. [30] conducted a literature review and consulted policy stakeholders to identify Australian collaborative care models for adults with severe mental illness (with a particular emphasis on models that addressed comorbidity, including AOD problems). One of their recommendations for enhancing collaboration was staff training in identifying and addressing comorbidity but also in respect to understanding the impact of comorbidity – a point reiterated by Sterling et al., [40] who also recommended training in how non-AOD providers refer to AOD services. Sylla et al. [39] echoed this call, but also concluded that, in the same way non-AOD clinicians could benefit from training in AOD issues, AOD clinicians need to be trained to identify and respond to broader life and wellbeing issues. They also highlighted that training needs to include guidance about roles and responsibilities in addressing issues beyond a clinician’s primary area of expertise. Training could also provide clinicians with an understanding of the different treatment models used by different agencies and how clients access care [30].

In addition, Lee et al. [30] highlighted the need to move beyond the model of training as an endpoint for implementation. They cited work highlighting the importance of ongoing coaching activities in reinforcing knowledge gained through training and in facilitating implementation. Such activities included providing consultation, supervision and case reviews, as well as facilitating partnership development between agencies and assisting agencies to manage change.

Urada et al. [29] highlight that training in AOD problems and treatment should also be incorporated into the curriculum of medical and nursing students, as well as social workers, psychologists and other care providers. Similarly Timko et al. [31] suggest that professional certification policies could encourage training in integrated care responses. This is likely to have a large impact on between-systems integration but is a long-term approach.

### Information sharing

Most forms of integrated care – whether co-located models, case-management or referral – require sharing of clinical and administrative information between agencies, clinicians and teams [30]. Gurewich et al. [38] found that primary care providers (referral sending agency) rarely received post-referral feedback from AOD services (referral recipient agency), and that often information was only relayed on to primary care providers by clients themselves in a relatively unsystematic way. This means that primary care providers may be unable to provide care consistent with care plans developed by AOD providers.

Client information can also be shared verbally through discussion at case conferences, in which clinicians from multiple agencies and multiple disciplinary backgrounds participate, or through team meetings where cases are discussed [30, 36, 41]. Vanderplaschen et al. [36] describe a systematic approach to verbal information sharing, which involves regular care coordination meetings between clinicians from different agencies involved in providing care to people with AOD problems. At each meeting, providers discussed 30 clients who are accessing two or more of the respective agencies in order to ensure a common strategy for addressing clients’ needs, monitoring progress and referrals provided. These care coordination meetings were perceived to be useful in terms of facilitating collaboration and knowledge exchange, as well as sharing information about each of the agency’s processes and treatment approaches. Re-imbursement of staff for attendance at care coordination meetings was considered important in making participation in meetings a routine component of clinicians’ roles – rather than a burdensome extra-curricular activity.

Based on the findings of their study, Lee et al. [30] recommended a number of other ways of sharing information, including sharing and developing combined care plans, undertaking assessments or case review meetings jointly, and making secondary consultation available so that providers can exchange knowledge. Lee et al. [30] also recommended developing client records that are in a format that can be relatively easily shared between different providers.

A number of studies [33, 38, 41] concluded that developing health Information Technology (IT) infrastructure compatible with the IT systems used in partner agencies could be beneficial in facilitating the sharing of clinical information. As well as having benefits in terms of space for storage, electronic client information systems are also likely to result in more timely information transfer. However, they can also be susceptible to security breaches, and one of the barriers to sharing information though electronic medical records were privacy issues [36].

### Case-management

Case-management was the most common model of service delivery identified as a useful approach to integrated care [33, 36]. Case-management has been found to be a particularly appropriate for clients with multiple and complex needs that cannot be met by a single provider [36]. In these cases, having a provider (e.g., case-manager) who can plan and arrange care can facilitate access and ensure needs are met [36]. In evaluating intensive case-management in Belgium, Vanderplaschen et al. [36] found that case-management also improved participation and retention in AOD treatment, and helped to avert crisis situations.

However, implementation can be challenging. Case-management is intensive and often requires a large time investment from case-managers, and sometimes clients can work with a case-manager for a long period of time, negatively impacting on the ability of case-managers to take on new clients [31]. Vanderplaschen et al. [31] recommended pooling resources from several treatment agencies to overcome some of the financial constraints of case-management. If relationships with other agencies don’t exist, case-managers can find themselves increasingly providing direct interventions to clients, which they may not have the capacity to do [31]. However, they argue that moving to a strength-based model of case-management and expanding sources of support to include informal help networks may overcome some of these challenges.

### Referral

Referral was identified by studies as a simple integrated working strategy that was useful when client needs were unable to be met by a clinician in a particular agency [32, 33, 38]. The likelihood of a clinician referring a client to another agency is not only dependent on the client need, but also on clinician’s perceptions of the quality of care provided at the referral-receiving agencies [32].

Based on the findings of their review, Lee et al. [30] recommended relaxing or removing exclusion criteria in terms of eligibility for clients to be accepted into a program as a way of enabling referral. It may not be feasible to remove all criteria for service delivery, but relaxing some criteria may be possible. For instance, some agencies have criteria about only accepting clients from a particular geographic area, which may be stipulated by their funder. If not stipulated by a funder, and if capacity exists, this is a criterion that agencies could relax.

### Development of inter-professional networks

While Scharf et al. [33] mentioned formalising inter-agency relationships, Sword et al. [32] identified that there is also an important role for more informal links between clinicians that can foster inter-agency relationships and act as pre-cursors to more formal partnerships. Indeed, one of the major barriers to integration is professional boundaries and territorialism [37], compounded by competitiveness between agencies.

The development of networks and joint events around shared interests are examples that Roberts [37] and Lee et al. [30] identified as ways of overcoming professional boundaries, and providing opportunities to share knowledge. Participants in Roberts’ [37] study recalled how groups advocating for better care for clients with AOD and mental health issues (dual diagnosis) and dual diagnosis projects (such as organising training, conferences, and lobbying) arose from the ground-up by interested clinicians, consumers and carers networking around a common purpose. However, the challenge with informal relationships developed between staff that do not materialise into formal relationships, is that they are sensitive to staff movements and departures.

## Level 4: clinical

Studies mentioned screening, routine informed consent practices, joint care planning and supervision as key approaches to improving integrated care at a clinical level. Many of these were found to be facilitated by service delivery and organisational level factors.

### Screening

Screening was one of the most widely mentioned clinical level strategies that could be implemented to enhance integration, particularly in relation to integration between AOD and non-AOD services. As Sylla et al. [39] concluded, screening for co-occurring problems is a good first step in improving integration, as it can enhance identification of problems and referral to other agencies. Similarly, Lubman et al. [42] document the successful adoption of mental health screening in a youth AOD service with 87.4% of eligible young people screened over a 30 month period. Successful implementation was underpinned by adequate training and management support, and embedding mental health screening within the services’ assessment form.

In the context of integrating AOD treatment into adolescent health care, Sterling et al. [40] recommended that screening using a “broad brush” approach is preferable to single-problem screening. They recognised that given the co-occurrence of AOD issues with a range of other health and social issues, trying to isolate AOD problems from this broader context is sub-optimal.

Developing common tools to support clinical decision making was one of the strategies mentioned by Kodner and Spreeuwenberg [12] in their outline of integrated working strategies. These are tools that are used across different agencies within a sector and sometimes between sectors. As more clinicians use a common screening tool for instance, a shared professional language is developed, which makes referral and information sharing easier. However, there is a need for training and support in using screening tools and interpreting their results, and how screening information can be used and shared to support referrals [42].

### Routine client consent to share information

Privacy issues were found to be a barrier to information sharing. However, studies have stressed the ethical and practical importance of routinely seeking client consent to share information before treatment commences [36, 41]. Lombard et al. [41] noticed variation in openness to sharing across agencies depending on their model of care, and the importance they attached to informed consent. At one agency that had an integrated approach, open communication between providers was encouraged, and client participation in the program was contingent on their agreeing to the sharing of information between multiple providers. Without informed consent to share information, clients were effectively excluded from integrated approaches such as case-management and care coordination [36].

### Joint care planning

Some studies not only discussed sharing care plans but jointly developing care plans. This is where two or more providers share information with each other in order to develop an optimal care plan that addresses the entirety of a client’s needs [41]. In some cases this was found to be aided by common care planning documentation [30], and care coordination meetings [36].

### Supervision

Two studies discussed how staff supervision could be important in enabling clinicians to undertake integrated care. One of these found that an external supervisor from a capacity building entity such as the Victorian Dual Diagnosis Initiative provided support to clinicians to implement dual diagnosis care [30]. Training providers may potentially play this kind of coaching role in the future. A more common form of supervision however, occurs between clinicians and supervisors (senior clinicians or team leaders) within an agency. Roberts [32] argues that workforce development strategies need to be accompanied by professional supervision, which is likely to facilitate the translation of learning into practice.

## Discussion

This review focussed on strategies to enhance integration in instances where at least one agency was an AOD treatment provider. While many of these have been identified as useful strategies in other health and social care contexts [12, 30, 43], our review highlights the way in which these manifest in the context of providing care to people with AOD problems. There are likely to be many more examples of integrated working practices and innovation in this area, but some may not have been documented, subjected to research or published in peer-reviewed journals. Indeed the small number of articles identified in the review is surprising given the emphasis placed on integrated AOD care in policy and practice. For instance, in Victoria, Australia a “No wrong door approach” policy has been in existence from 2007 and specifies that people who present to AOD services with co-occurring mental health problems should be welcomed, and that people with co-occurring AOD problems should also be able to receive appropriate care in mental health services [44].

The approach that researchers have taken in studying integrated care has been to examine a number of strategies occurring concurrently. It is almost impossible to isolate a single strategy and study its effectiveness, because of the embedded nature of partnership and integration. This is perhaps why we did not find any randomised control trials of strategies to improve integrated care to people with AOD problems. However, the lack of experimental studies also means that we are unable to ascertain which kinds of integrated working strategies are more likely to be efficacious than others given particular sets of circumstances. As is appropriate in this kind of health services and systems research, studies often utilised qualitative studies, case studies involving rich descriptions of strategies and contexts, and mixed method approaches. Despite the employment of study designs that do not lend themselves toward generalisability, many articles contained recommendations for broader implementation, which is a reason to exercise caution in interpreting the results of our review. The lack of longitudinal studies identified means that it is unclear if, and how integrated care improvements were sustained. It also means that our review was unable to ascertain the degree to which particular integrated working strategies contributed to client treatment outcomes and levels of satisfaction with treatment.

With the exception of one study [36], all studies focussed on strategies to enhance integration between AOD and non-AOD services. Further work is needed to examine strategies to improve integration between AOD services as well as factors contributing to sustained improvements over time. Similarly, as all studies were from North America, Europe or Australia, there is a need to explore strategies for enhancing integrated care in other settings. Furthermore, one of the limitations of our review was that it did not include articles and reports that were not published in peer-reviewed academic journals. If it had, a broader range of strategies for facilitating integrated care may have been identified.

Despite these limitations in the literature and of our review, our findings echo the need to focus on implementing packages of strategies [12] rather than a single strategy undertaken in isolation or as a one-off. The themes we identified were so interconnected that they often operated across levels of care, and impacted on other strategies. For instance, referral as a strategy was found to be enhanced by clinician training in how to identify a broad range of needs and how to refer, use of common clinical tools that create a shared professional language, information sharing, and the quality of inter-agency relationships. Having said this, inter-agency relationships appear to impact on most other strategies, reinforcing the importance of these relationships to integrated working [45]. Thus, there is a need for systems intervention approaches that are adequately funded rather than those undertaken solely within agencies.

Based on the results of the review, we propose a number of preliminary suggestions for enhancing integrated care for people with AOD and other co-occurring problems (please see Additional file 3). Given the aforementioned limitations of the literature in terms of the type of studies, generalisability, and lack of information on the effectiveness of integrated working strategies, these are simply ideas that will need empirical testing. The appropriateness of each suggestion and the way in which they are implemented as part of a package of strategies will depend on the partnership and agency context. All will require coordination, partnership, an implementation strategy and an evaluation approach that allows reflexive consideration of success and lessons learnt.

Agencies will need to prioritise which package of strategies is appropriate to their context. We therefore propose a stepwise approach for implementation of integrated working strategies that will guide urgent requirements and key priorities. The process includes four steps and a series of tools which can be accessed online (via www.turningpoint.org.au):

- Step 1: Baseline assessment - Given that agencies and partnerships operate in different contexts, our first recommendation is that agencies map existing inter-agency relationships to identify needs and priorities. Having conducted a baseline assessment of integrated working, agencies will be well placed to select which packages of strategies to implement.
- Step 2: Goal setting and implementation planning –The next step is to set integrated working goals based on information gathered during baseline assessment and decide how to implement strategies. It can be useful to develop an implementation plan to document actions, timeframes and expected outcomes.
- Step 3: Piloting and rollout of strategies – Piloting strategies before scaling up implementation across an agency is a wise approach. Not only does piloting provide an opportunity to test and refine the strategies on a small scale, it also enables time for staff, clients and carers to adjust to change.
- Step 4: Monitoring, evaluation and dissemination - In order to ascertain whether integrated working strategies have been successful, and how they could be strengthened, monitoring and evaluation are needed. This can occur through collecting data on clinician as well as client and carer perspectives. Findings of evaluation should be disseminated to key stakeholders so that lessons and innovative approaches can be shared.

Further work is needed to empirically test this model. In addition, local level needs assessment of integrated working is needed as strategies will need to be tailored to context.

## Conclusions

Despite research highlighting the benefits of integrated care, few studies have examined what strategies agencies can implement to improve integrated working in the context of providing care to people with AOD problems. Although there are considerable limitations in the current evidence-base, this systematic review of the literature draws attention to the need for a multi-pronged and multi-level approach to improving integrated care. As well as deriving preliminary suggestions for improved integrated care based on the findings, the proposed method for guiding implementation of integrated care strategies may be useful for policy makers, service managers and clinicians.

## Additional files

Additional file 1: Levels of integrated care strategies (adapted from Kodner & Spreeuwenberg [12], and Kodner & Kryiacou [46]). (DOCX 21 kb)

Additional file 2: Summary of included study characteristics, findings and integrated working strategies. (DOCX 23 kb)

Additional file 3: Preliminary suggestions. (DOCX 18 kb)

## Abbreviations

AOD: Alcohol and other drug
BH: Behavioural health
CHCs: Community health centres
HIV: Human immunodeficiency virus
IT: Information technology
MOU: Memoranda of understanding
OSATs: Outpatient substance abuse treatment
SAMHSA: Substance abuse and mental health services administration
TB: Tuberculosis
USA: United States of America

## References

[1] Barker SF, Best D, Manning V, Savic M, Lubman DI, Rush B (2016). A tiered model of substance use severity and life complexity: potential for application to needs-based planning. Subst Abus.

[2] Lubman DI, Garfield JB, Manning V, Berends L, Best D, Mugavin J (2016). Characteristics of individuals presenting to treatment for primary alcohol problems versus other drug problems in the Australian patient pathways study. BMC Psychiatry.

[3] Manning V, Garfield JB, Best D, Berends L, Room R, Mugavin J (2016). Substance use outcomes following treatment: findings from the Australian patient pathways study. Aust N Z J Psychiatry.

[4] Treloar C, Holt M (2008). Complex vulnerabilities as barriers to treatment for illicit drug users with high prevalence mental health co-morbidities. Ment Health Subst Use.

[5] Gilchrist G, Radcliffe P, Noto AR, d’Oliveira AFPL (2016). The prevalence and factors associated with ever perpetrating intimate partner violence by men receiving substance use treatment in Brazil and England: a cross-cultural comparison. Drug Alcohol Rev.

[6] Laudet AB, Stanick V, Sands B (2009). What could the program have done differently? A qualitative examination of reasons for leaving outpatient treatment. J Subst Abuse Treat.

[7] Sterling S, Chi F, Hinman A (2011). Integrating care for people with co-occurring alcohol and other drug, medical, and mental health conditions. Alcohol Res Health.

[8] VAGO (2011). Managing drug and alcohol prevention and treatment services.

[9] Radcliffe P, Gilchrist G (2016). You can never work with addictions in isolation: addressing intimate partner violence perpetration by men in substance misuse treatment. Int J Drug Policy.

[10] Armitage GD, Suter E, Oelke ND, Adair CE (2009). Health systems integration: state of the evidence. Int J Integr Care.

[11] Leichsenring K (2004). Developing integrated health and social care services for older persons in Europe. Int J Integr Care.

[12] Kodner DL, Spreeuwenberg C (2002). Integrated care: meaning, logic, applications, and implications - a discussion paper. Int J Integr Care.

[13] Neale J, Nettleton S, Pickering L (2011). What is the role of harm reduction when drug users say they want abstinence?. Int J Drug Policy.

[14] Futterman R, Lorente M, Silverman S (2004). Integrating harm reduction and abstinence-based substance abuse treatment in the public sector. Subst Abus.

[15] Marlatt GA, Blume AW, Parks GA (2001). Integrating harm reduction therapy and traditional substance abuse treatment. J Psychoactive Drugs.

[16] Denning P (2001). Strategies for implementation of harm reduction in treatment settings. J Psychoactive Drugs.

[17] Hunt GE, Siegfried N, Morley K, Sitharthan T, Cleary M (2013). Psychosocial interventions for people with both severe mental illness and substance misuse. Cochrane Database Syst Rev.

[18] Drake RE, O’Neal EL, Wallach MA (2008). A systematic review of psychosocial research on psychosocial interventions for people with co-occurring severe mental and substance use disorders. J Subst Abuse Treat.

[19] Donald M, Dower J, Kavanagh D (2005). Integrated versus non-integrated management and care for clients with co-occurring mental health and substance use disorders: a qualitative systematic review of randomised controlled trials. Soc Sci Med.

[20] Schulte SJ, Meier PS, Stirling J (2011). Dual diagnosis clients’ treatment satisfaction - a systematic review. BMC Psychiatry.

[21] Weisner C, Mertens J, Parthasarathy S, Moore C, Lu Y (2001). Integrating primary medical care with addiction treatment: a randomized controlled trial. JAMA.

[22] Willenbring ML, Olson DH (1999). A randomized trial of integrated outpatient treatment for medically ill alcoholic men. Arch Intern Med.

[23] Saxon AJ, Malte CA, Sloan KL, Baer JS, Calsyn DA, Nichol P (2006). Randomized trial of onsite versus referral primary medical care for veterans in addictions treatment. Med Care.

[24] Andersen M, Paliwoda J, Kaczynski R, Schoener E, Harris C, Madeja C (2003). Integrating medical and substance abuse treatment for addicts living with HIV/AIDS: evidence-based nursing practice model. Am J Drug Alcohol Abuse.

[25] Chi FW, Parthasarathy S, Mertens JR, Weisner CM (2011). Continuing care and long-term substance use outcomes in managed care: early evidence for a primary care-based model. Psych Serv.

[26] Milligan K, Niccols A, Sword W, Thabane L, Henderson J, Smith A, Liu J (2010). Maternal substance use and integrated treatment programs for women with substance abuse issues and their children: a meta-analysis. Subst Abuse Treat Prev Policy.

[27] Morgenstern J, Hogue A, Dauber S, Dasaro C, McKay JR (2009). A practical clinical trial of coordinated care management to treat substance use disorders among public assistance beneficiaries. J Consult Clin Psychol.

[28] Moher D, Liberati A, Tetzlaff J, Altman DG (2009). Preferred reporting items for systematic reviews and meta-analyses: the PRISMA statement. Ann Intern Med.

[29] Urada D, Teruya C, Gelberg L, Rawson R (2014). Integration of substance use disorder services with primary care: health center surveys and qualitative interviews. Subst Abuse Treat Prev Policy.

[30] Lee SJ, Crowther E, Keating C, Kulkarni J (2013). What is needed to deliver collaborative care to address comorbidity more effectively for adults with a severe mental illness?. Aust N Z J Psychiatry.

[31] Timko C, Valenstein H, Lin PY, Moos RH, Stuart GL, Cronkite RC (2012). Addressing substance abuse and violence in substance use disorder treatment and batterer intervention programs. Subst Abuse Treat Prev Policy.

[32] Sword W, Niccols A, Yousefi-Nooraie R, Dobbins M, Lipman E, Smith P (2013). Partnerships among Canadian agencies serving women with substance abuse issues and their children. Int J Ment Health Addict.

[33] Scharf DM, Eberhart NK, Schmidt N, Vaughan CA, Dutta T, Pincus HA, Burnam MA (2013). Integrating primary care into community behavioral health settings: programs and early implementation experiences. Psych Serv.

[34] Drabble L (2007). Pathways to collaboration: exploring values and collaborative practice between child welfare and substance abuse treatment fields. Child Maltreat.

[35] Lindholm M, Ryan D, Kadushin C, Saxe L, Brodsky A (2004). “Fighting back” against substance abuse: the structure and function of community coalitions. Hum Organ.

[36] Vanderplasschen W, Mostien B, Franssen A, Lievens K, De Maeyer J, Broekaert E (2007). Dealing with multiple and frequent service utilisation in substance abuse treatment: Experiences with coordination of care in residential substance abuse agencies in the region of Ghent, Belgium. Ther Communities.

[37] Roberts B (2012). Interprofessional relationships in dual diagnosis discourse in an Australian state: are we respecting each other yet?. Ment Health Subst Use.

[38] Gurewich D, Prottas J, Sirkin JT (2014). Managing care for patients with substance abuse disorders at community health centers. J Subst Abuse Treat.

[39] Sylla L, Bruce RD, Kamarulzaman A, Altice FL (2007). Integration and co-location of HIV/AIDS, tuberculosis and drug treatment services. Int J Drug Policy.

[40] Sterling S, Valkanoff T, Hinman A, Weisner C (2012). Integrating substance use treatment into adolescent health care. Curr Psychiatry Rep.

[41] Lombard F, Proescholdbell RJ, Cooper K, Musselwhite L, Quinlivan EB (2009). Adaptations across clinical sites of an integrated treatment model for persons with HIV and substance abuse. AIDS Patient Care STDs.

[42] Lubman DI, Hides L, Scaffidi A, Elkins K, Stevens M, Marks R (2008). Implementing mental health screening within a youth alcohol and other drugs service. Ment Health Subst Use.

[43] Fuller JD, Perkins D, Parker S, Holdsworth L, Kelly B, Fragar L (2009). Systematic review on service linkages in primary mental health care: informing Australian policy and practice.

[44] Roberts BM, Maybery D (2014). Dual diagnosis discourse in Victoria Australia: the responsiveness of mental health services. J Dual Diagn.

[45] Carter WC, Lee SYD, Thomas KC, Morrissey J (2006). Managed care, inter-agency linkages, and outpatient substance abuse treatment. Adm Policy Ment Health.

[46] Kodner DL, Kyriacou CK (2000). Fully integrated care for frail elderly: two American models. Int J Integr Care.

