# Supplementary material for: Strategies to facilitate integrated care for people with alcohol and other drug problems: a systematic review
Source: Subst Abuse Treat Prev Policy. 2017 Apr 7;12:19. doi: 10.1186/s13011-017-0104-7 (PMC5384147; doi:10.1186/s13011-017-0104-7)
Supplement: Supplementary file 1 — Levels of integrated care strategies (adapted from Kodner & Spreeuwenberg [12], and Kodner & Kryiacou [46]). (DOCX 21 kb) [file 13011_2017_104_MOESM1_ESM.docx]

*Additional file 1: Levels of integrated care strategies (adapted from Kodner & Spreeuwenberg [12], and Kodner & Kryiacou [45])*

| **Level** | **Definition** | **Strategies** | **Explanation** |
| --- | --- | --- | --- |
| Funding | How health and social care funds are divided and the way in which services are funded affects how they operate and the degree to which they provide integrated care. | Pooling of funds (at various levels)  Prepaid capitation (at various levels)  Providing funding over longer time windows to improve security and increase opportunities for relationship building  Performance management targets that focus on integration | Contributions are pooled so that the costs of health care are shared by all and not borne by individuals at the time they fall ill (this requires a certain level of solidarity)  Payment the state agency makes periodically to a contractor on behalf of each beneficiary enrolled under a contract for the provision of medical services under the state plan. |
| Administrative | Government regulatory and administrative departments play a role in integrated care, as they have a crucial role in system design and stewardship. Inter-sectoral planning for example may result in a better vertically integrated system that is able to accommodate the multiple needs of clients. | Consolidation and/or decentralisation of responsibilities and/or functions  Inter-sectoral planning  Needs assessment/allocation chain  Joint purchasing or commissioning  Performance management | Transfer of authority and power from higher to lower levels of government or  system that helps to understand , assess and meet the local needs, which in turn increases greater sustainability of positive outcomes  Creation of a shared vision of  the future, identification and ranking of key issues, development of action plans,  mobilization of resources and ways of increasing public support among providers  Mechanisms to ensure clients receive the “right care at the right place at the right time”. This requires a thorough understanding of the way in which clients move within and between different health and social care providers  Activities involved in assessing and forecasting needs, links investment to agreed desired outcomes, considering options, planning the nature, range and quality of future services and working in partnership to put these in place undertaken by two or more organisations working together.    A process for using data to improve care delivery and outcomes and to measure the effectiveness of care |
| Organisational | Organisations that provide health and social care can improve integration through formally and informally networking, collaborating with other agencies, and joint working. Joint working strategies can include multi-agency teams and co-location of staff in which staff from one agency are placed in another agency for a day a week for instance. Here, senior managers play a crucial role in building and sustaining shared culture and values; maintaining oversight of pooled resources and funding  streams; coordinating joint targets; supervising diverse staff; managing complex organisational structures and relationships. | Co-location of services  Discharge and transfer agreement  Inter-agency planning and/or budgeting  Service affiliation or contracting  Jointly managed programs or services  Strategic alliances or care networks  Consolidation, common ownership or merger  Workforce development and planning | Strategies that place multiple services in the same physical space.  Written health facility agreement with one or more other services or health care institutions, for the transfer or discharge of clients from one to another, along with the orderly exchange of relevant clinical information about the clients transferred or discharged.  Overall responsibility for total quality and costs of services  A professional relationship including an employment relationship, a position as an independent contractor or granting of privileges by a health care facility  Ability to bundle and access a broad range of needed health and social care services from anywhere on the continuum of care  Nature of working arrangements among and between institutions and service providers  Consolidation can occur through either merger or acquisition Mergers—transactions in which separate services come together under a shared license—typically occur among services located near one another. Acquisitions occur when joining services retain their licenses but are owned by a common governing body; they can occur among services that are near or far away. |
| Service delivery | How staff are trained, deliver care, relate to their clients and colleagues, and how they work together has an impact on the clients direct experience of integrated care. Staff trained in case-management may be well equipped to deliver integrated care whereas those that aren’t may be less well equipped. | Joint training – workforce development  Centralised information, referral & intake  Case/care management delivery model  Multidisciplinary/interdisciplinary teamwork  Around-the-clock (on-call) coverage  Integrated information systems | Training across professional groups that creates new varieties of health care worker.  Access to the full suite of specialty services in the most timely and equitable way  Planning, arrangement and monitoring of needed care across time, place and discipline  Ongoing communication and collaboration among, and clinical management by, a multidisciplinary group of providers.  Being available to provide timely access to care to clients after-hours.  Access to and use of shared clinical data including treatment protocols, administrative, and financial information on a manual and/or automated basis |
| Clinical | At the micro or clinical level a common professional language and tools (such as screening and assessment tools) as well as practice standards can facilitate integrated care and embed a holistic way of working in everyday practice. | Standard diagnostic criteria  Uniform, comprehensive assessment procedures  Uniform comprehensive screening procedures  Joint care planning  Shared clinical record(s)  Continuous patient monitoring  Common decision support tools (e.g . practice guidelines and protocols, including shared assessment and review documentation)  Regular patient/family contact and ongoing support | Compliance to agreed standard diagnostic criteria  Commitment to performing comprehensive, multidimensional patient evaluation  Commitment to performing comprehensive patients screens to make timely referral to partner agencies  Control over transitions between benefits, settings and providers  Share information on the clinical care and health status of an individual or group of people.  Regular measurement, review and record of clients progress and wellbeing  Use of common and agreed ways of representation of health knowledge  Continuous planning, delivery, and evaluation of health care that is grounded in mutually beneficial partnerships among health care providers, patients, and  families |
