# Supplementary material for: Strategies to facilitate integrated care for people with alcohol and other drug problems: a systematic review
Source: Subst Abuse Treat Prev Policy. 2017 Apr 7;12:19. doi: 10.1186/s13011-017-0104-7 (PMC5384147; doi:10.1186/s13011-017-0104-7)
Supplement: Supplementary file 2 — Summary of included study characteristics, findings and integrated working strategies. (DOCX 23 kb) [file 13011_2017_104_MOESM2_ESM.docx]

*Additional file 2: Summary of included study characteristics, findings and integrated working strategies*

| ***Study authors and year*** | ***Study aim*** | ***Study type*** | ***Setting*** | ***Target population*** | ***Type of integration*** | ***Integrated working strategies studied/identified*** |
| --- | --- | --- | --- | --- | --- | --- |
| 1.Scharf et al., [33] | To describe the integration of primary care services into community behavioral health settings based on review of Substance Abuse and Mental Health Services Administration (SAMSHA) Primary and Behavioral Health Care Integration grants. | Data extraction from grantee proposals from 56 programs, across  26 states. | Unites States of America (USA) | Adults with serious mental illness and AOD use. | Between primary care organization and behavioral care organization. | Nurse care management, co-location of nurse practitioners and primary care providers, supervision by primary care providers |
| 2. Sword et al., [32] | To determine predictors that influence partnership among agencies for women with AOD problems. | Online survey of 270 agencies. | Canada | Women with AOD problems. | Responding agencies most frequently partnered with social agencies, followed by health care and child agencies; they least frequently partnered with prenatal/postnatal services and universities. | Partnership activities; referrals sent; referrals received; information shared;  consultation; joint programs. |
| 3. Lee et al., [30] | To identify collaborative care models for adults with comorbidities involving severe mental illness. | Literature review and consultation with national stakeholders. | Australia | Clients with severe mental illness and AOD problems. | Between mental health treatment and AOD services. | Exchange client information, shared treatment plans, joint case conferences, consultations, comorbidity screen­ing and staff education. |
| 4. Sterling et al., [40] | To examine the literature on integration of AOD treatment with other adolescent health care services focusing on Screening, Brief Intervention and Referral to  Treatment. | Literature review. | USA | Adolescents with AOD problems and other issues. | Between AOD services and Emergency Departments, primary care, and school-based health care centers. | Delivery of brief intervention in emergency department settings, physician-delivered risk behavior screening and counseling; access to behavioral health services, AOD treatment integration into college health centres. |
| 5.Vanderplasschen et al., [36] | To examine the impact of inter-agency care coordination and intensive case-management on multiple service utilization. | Qualitative component of a larger randomized controlled trial of case managers/care coordinators and 20 clients. | Belgium | Adults with substance abuse in residential treatment. | Between detoxification AOD services and outpatient services. | Case-management |
| 6. Roberts [37] | To explore the attitudes of specialist mental health workers to AOD problems in the process of developing effective services for people with ‘dual diagnosis’. | Qualitative case study of 18 policy advisors, consumer researchers  and clinicians. | Australia | Clients with co-occurring AOD and mental health (dual diagnosis) issues. | Between AOD and mental health services. | Development of new community-based service system in Victoria. |
| 7. Gurewich et al., [38] | To examine operational practices of community health centres (CHCs) to effectively coordinate substance use disorders. | Case study of 25 staff from 5 CHCs. | USA | Adults with AOD problems. | Between CHCs and AOD service. | Co-location of services, linkage of workers. |
| 8. Carter et al., [46] | To investigate how managed care  arrangements affect inter-agency relationships of  outpatient substance abuse treatment (OSATs) with primary care and mental health agencies. | Cross-sectional survey of 62  OSATs. | USA | Adults with AOD problems. | Between AOD, primary care and mental health services. | Managed care arrangements. |
| 9. Lombard et al., [41] | To examine the integration of Human Immunodeficiency Virus (HIV) and AOD care. | Qualitative study | USA | Adults with HIV and AOD problems and mental illness. | Between AOD and HIV care. | Case-management |
| 10. Sylla et al., [39] | To examine integration of three clinical services:  - Screening, detection and treatment of patients with tuberculosis (TB).  - Voluntary Counselling, Testing and medical management for HIV clients.  - Screening and treatment of substance use and dependence. | Literature review. | USA | Adults with HIV/TB and AOD problems. | Between AOD, TB and HIV care. | Case-management, co-location, cross-training |
| 11. Lindholm et al., [35] | To describe possibilities of community coalition to reduce AOD problems in 10 urban communities. | Qualitative study (e.g.. case histories) of 8 programs. | USA | Adults with AOD problems. | Between AOD and community agencies and groups. | Community coalitions |
| 12. Urada et al., [29] | To examine AOD service integration in primary care. | Mixed methods study: survey of primary care service staff and qualitative interviews with managers, clinicians, and clients. | USA | Primary health care centres. | Between AOD and primary care services. | Provider funding, training, expanding AOD treatment services, co-location of AOD services in primary care. |
| 13. Lubman et al., [42] | To describe the implementation and adoption mental health screening within a youth AOD service in Australia. | Quantitative examination of routinely collected service data. | Australia | Young people with AOD and mental health problems. | Between AOD and mental health services. | Screening |
| 14. Timko et al., [31] | To examine the extent to which AOD services address intimate partner violence and batterer intervention programs address AOD problems. | Quantitative survey of directors of AOD services (n=241) & batterer intervention programs (n=235). | USA | People with AOD problems who have perpetrated intimate partner violence. | Between AOD and intimate partner violence services. | Assessment, referral, care-planning, cross-training, service centralization, & programs focussing on co-occurring issues. |
