# Supplementary material for: Strategies to facilitate integrated care for people with alcohol and other drug problems: a systematic review
Source: Subst Abuse Treat Prev Policy. 2017 Apr 7;12:19. doi: 10.1186/s13011-017-0104-7 (PMC5384147; doi:10.1186/s13011-017-0104-7)
Supplement: Supplementary file 3 — Preliminary suggestions. (DOCX 18 kb) [file 13011_2017_104_MOESM3_ESM.docx]

*Additional file 3: Preliminary suggestions*

1. Funding of integrated working is needed and this should be included in service specifications across government departments to ensure that integrated working is core business. Without this, there may be little time, resources and incentive to implement integrated working strategies. Once this system level approach has been adopted there are a number of strategies that can be implemented by agencies and clinicians. There also needs to be clear processes for monitoring the effectiveness of integrated working at a systems level as well as at a service level.
2. At the organisational level, mapping of inter-agency relationships as outlined by Sword et al., [32] is a promising way of identifying where new partnerships are needed, and where existing relationships could be strengthened. Formalising agency relationships through MOUs, letters, and formal contracts can ensure agency commitment to partnership and accountability, especially if expectations and goals are documented. Agencies need to have governance structures for managing partnerships and conflict that may arise. Partnership advisory groups headed by a neutral convener and incorporating consumer and carer input, might be a good option.
3. The development of professional interest groups, networks and events that include professionals from a range of agencies can facilitate knowledge exchange and informal relationships between providers that may blossom in to formal relationships. These sorts of initiatives need to be fostered, and grown.
4. Clinician training is a useful way of increasing capacity to provide integrated responses. Where possible, training should address identifying and responding to issues that affect or affected by AOD, roles and responsibilities of clinician’s in addressing issues beyond their primary areas of expertise, referring to AOD and non-AOD agencies and the treatment models these agencies operate under, the implementation of integrated working practices. Integrated working could be enhanced if training providers supplement single training session models with a focus on longer term implementation coaching and capacity building. For instance, training providers could also provide follow-up visits and workshop implementation barriers, consultation on integrated working practice, supervision and case reviews, and assistance evaluating integrated working strategies.
5. Information sharing is also critical to most integrated care responses. This can be facilitated by the implementation of routine processes to seek informed client consent for sharing of information, ensuring client records are in a format that can be easily shared (e.g., electronic, pdfs etc.), and by ensuring the compatibility of health IT infrastructure between partner agencies (particularly between AOD agencies).
6. Co-location of services can be a potentially useful integrated working strategy that agencies could implement. However, physical space issues, ways in which to coordinate care and share information between the co-located team/clinician and the host agency, and staff workload issues will need to be considered prior to implementation.
7. Case-management service delivery models are likely to be particularly appropriate for clients with multiple and complex needs. Due to the intensity of this approach, agencies adopting case-management may benefit from strengths based approaches, and may like to consider widening the net of sources of support to also include community groups to whom clients could be linked. Regular case or care coordination meetings can be a useful strategy to facilitate communication between providers for clients who have involvement in a number of services. It can also ensure the needs of clients are being met and that they are not falling through the gaps. To ensure the sustainability and effectiveness of this process, participation in regular care coordination meetings needs to be included in the role of providers, who need to be adequately reimbursed for this activity.
8. At the clinical level, the implementation of common AOD screening and assessment tools and/or tools used in non-AOD agencies may aid early identification of issues and facilitate information sharing, and referral. Similarly, sharing and jointly developing care plans between agencies can ensure that approaches to responding to needs are holistic and are complementary rather than being at odds or duplicating care.
